# Metformin revert insulin‐induced oxaliplatin resistance by activating mitochondrial apoptosis pathway in human colon cancer HCT116 cells

**DOI:** 10.1002/cam4.3029

**Published:** 2020-04-05

**Authors:** Chao Liu, Qianqian Liu, Aiwen Yan, Hui Chang, Yuyin Ding, Junye Tao, Chen Qiao

**Affiliations:** ^1^ Department of Pharmacy Nanjing First Hospital Nanjing Medical University Nanjing Jiangsu China

**Keywords:** AMPK, insulin, metformin

## Abstract

**Background:**

Several studies have suggested that drug resistance in colon cancer patients with diabetes may be associated with long‐term insulin administration, which in turn decreases the survival rate. Metformin is a commonly used drug to treat diabetes but has been recently demonstrated to have a potential therapeutic effect on colon cancer. This study aimed to elucidate the underlying mechanism by which metformin reverts insulin‐induced oxaliplatin resistance in human colon cancer HCT116 cells.

**Methods:**

Two colon cancer cell lines (HCT116 and LoVo) were used to verify whether the expression of insulin receptor substrate 1 (IRS‐1) could impact the half maximal inhibitory concentration (IC50) of oxaliplatin after chronic insulin treatment. The IC50 of oxaliplatin in both cell lines was measured to identify metformin sensitization to oxaliplatin. The adenosine monophosphate‐activated protein kinase (AMPK) inhibitor, namely AMPK small interfering RNA, was used to block AMPK activation to identify critical proteins in the AMPK/Erk signaling pathway. Bcl‐2 is a vital antiapoptotic protein involved in the mitochondrial apoptosis pathway. Finally, immunofluorescence and electron microscopy were performed to investigate how metformin affects the ultrastructural integrity of mitochondria.

**Results:**

The IC50 of oxaliplatin in HCT116 cells was noticeably increased. After the cells were treated with metformin, oxaliplatin resistance was reversed. According to the results of the western blotting assay of vital proteins involved in the classical apoptosis pathway, cleaved caspase‐9 was noticeably upregulated, suggesting that the mitochondrial apoptosis pathway was activated. These results were verified by imaging of mitochondria using electron microscopy. The AMPK/Erk signaling pathway experiments revealed that the upregulation of Bcl‐2 induced by insulin through Erk phosphorylation was inhibited by metformin and that such inhibition could be mitigated by the inhibition of AMPK.

**Conclusions:**

Insulin‐induced oxaliplatin resistance was reversed by metformin‐mediated AMPK activation. Accordingly, metformin is likely to sensitize patients with diabetes to chemotherapeutic drugs used to treat colon cancer.

## Graphical abstract

Our studies in chronic insulin‐treated HCT‐116 cells suggested that metformin could inhibit Bcl‐2 expression, and its mitochondrial translocation to facilitate mitochondrial apoptosis via AMPK/Erk signaling pathway.

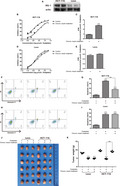

## 1 BACKGROUND

Colorectal cancer (CRC) has become the third most common gastrointestinal cancer in the world and younger patients are increasingly being diagnosed with CRC.^1^ Diabetes is considered to be a vital prognostic factor of CRC, and according to numerous studies, the risk for CRC is closely related to that for diabetes.^2^ Insulin resistance refers to a pathological condition resulting from chronic hyperinsulinemia. One of the underlying molecular mechanisms of this adaptive process involves a shift in the phosphorylation status of the insulin receptor substrate 1 (IRS‐1). This shift may trigger numerous downstream signaling pathways, such as extracellular signal‐regulated kinase (Erk), which promote cell proliferation, cell survival, and enhancement of chemotherapeutic tolerance in cancer. For example, chronic hyperinsulinemia can induce chemoresistance, especially to oxaliplatin. In patients with CRC,^3^ long‐term insulin treatment has been shown to be associated with poor prognosis in those undergoing chemotherapy.^4^

Metformin, a common oral drug used to treat hypoglycemia, exerts an anticancer effect both in vivo and in vitro.^5^ Epidemiological studies have also suggested that patients administered metformin have a lower risk for cancer.^6^ Results of a previous meta‐analysis suggested that metformin could increase the survival rate of CRC patients with type II diabetes mellitus.^7^ Metformin can prevent hyperinsulinemia‐induced adverse prognosis in chemotherapy for digestive tract cancer (eg, CRC).^8^ Metformin is an adenosine monophosphate‐activated protein kinase (AMPK) capable of inhibiting Erk activation and apoptosis in cancer cells. Other studies have also verified that with the intact AMPK signaling axis, metformin can inhibit the Warburg effect, which demonstrates a specific energy metabolism pathway in cancer.^9^

Collectively, these data suggest that metformin, a first‐line, oral anti‐diabetes drug, may exhibit anticancer activity by regulating signaling pathways downstream of AMPK. However, the exact role of metformin in inducing AMPK‐mediated apoptosis remains unclear. In the present study, chemotherapy sensitization to oxaliplatin in patients with colon cancer cell line HCT116 was tested.

## 2 MATERIALS AND METHODS

### 2.1 Materials and cell culture

The human CRC cell lines, HCT116 and LoVo, were purchased from the Cell Bank of Shanghai Institute of Biochemistry and Cell Biology, Chinese Academy of Sciences, which were cultured in Dulbecco's modified Eagle medium (Gibco TM, 11965092) with 10% fetal bovine serum (Biological Industries, 04‐001‐1A‐US), 100 U/mL penicillin, and 100 mg/mL streptomycin (Gibco TM, 15140‐122) at 37°C in an atmosphere of 5% CO_2_. Oxaliplatin (O9512), metformin (PHR1084), insulin (1342106), AMPK inhibitor, and compound C (171260) were purchased from Sigma‐Aldrich. LY3214996 was purchased from MedChem Express. AMPK siRNA was purchased from Santa Cruz Biotechnology. Cell Counting Kit‐8 (KGA317s‐500) was purchased from KeyGen. For transfection, HCT116 cells were seeded in 6‐well plates at 70% confluency, and then transfected with AMPK siRNA (5 nmol/L) using Lipofectamine 2000 (Invitrogen/Thermo‐Fisher Scientific) according to the manufacturer's instructions. The knockdown efficiency at the optimized concentration was < 1%.

### 2.2 Cell viability

The chronic insulin treatment model was established by 12 weeks of insulin (20 nmol/L) treatment. To evaluate the sensitivity of oxaliplatin in tumor cells, cell viability was analyzed using the CCK‐8 assay. Cells growing in the logarithmic phase were collected and seeded into 96‐well plates at a concentration of 5 × 10^3^ cells per well and cultured overnight. After cell attachment, varying concentrations of oxaliplatin and metformin were added, and the cells were cultured for an additional 24 hours. Subsequently, CCK‐8 was added to each well, followed by incubation for an additional 4 hours at 37°C. Absorbance (optical density) was measured using a microplate reader (Thermo Multiclan) at a wavelength of 450 nm. The results are expressed as the mean ± standard error of the mean (SEM) (n = 6 for each group) inhibition rate of cells compared with normal controls.

### 2.3 Animal model

Forty athymic male BALB/c nude mice (age, 35‐40 days; weight, 18‐22 g) were obtained from the Experimental Animal Center of Zhejiang Province. The mice were housed at ambient temperature (25 ± 5°C) and 45 ± 5% relative humidity with a 12‐hours light/dark cycle (lights on from 06:00 to 18:00), a standard diet containing 20% w/w fat, and ad libitum access to water. The tumor xenograft model was established by injecting 100 μL of D‐Hank's containing 5 × 10^6^ cells into the right axilla after the mice acclimatized for 1 week. After 12 days of growth, mice exhibiting similar tumor volumes were randomly divided into six groups (6 mice/group), as follows: LoVo control; oxaliplatin (5 mg/kg [oxaliplatin‐LoVo]); chronic insulin + oxaliplatin (5 mg/kg [chronic insulin + oxaliplatin‐LoVo]); HCT116 control; oxaliplatin (5 mg/kg [oxaliplatin‐HCT116]); and chronic insulin + oxaliplatin (5 mg/kg [chronic insulin + oxaliplatin‐HCT116). All experimental procedures conformed to the Guide for Care and Use of Laboratory Animals of the Chinese National Institutes of Health. All experiments were approved by the Nanjing First Hospital Ethics Committee (KY 20190302‐07).

### 2.4 Western blot assay

Cells were harvested and then lysed using 200‐μL ice‐cold RIPA lysis buffer (KeyGEN BioTECH, KGP702‐100) consisting of 1 mmol/L PMSF and protease inhibitor cocktail. A commercially available BCA protein assay kit (KeyGEN BioTECH, KGP902) was used to quantify proteins in the lysate. Cell lysates consisting of 100‐µg protein were loaded onto a sodium dodecyl sulfate polyacrylamide gel with an 8%‐12% gradient. After transferring the proteins to polyvinylidene membranes, they were detected using immunoblotting methods with antibodies against the relevant proteins, as follows: antibody to β‐actin (BM0627) was obtained from Boster (Wuhan, China); caspase‐8 (CST, #4927), caspase‐9 (CST, #9504), AMPKα (CST, #2532), Phospho‐AMPKα (Thr172) (CST, #2531), p44/42 MAPK (Erk1/2) (CST, #9102), Phospho‐p44/42 MAPK (Erk1/2) (Thr202/Tyr204) (CST, #4370), IRS‐1 (CST, #2382), and Phospho‐IRS‐1 (Ser307) (CST, #2381) were procured from Cell Signaling Technology; COX IV (AB3119), Bcl‐2 (AB182858), Caspase‐3 (AB13847), AIF (AB1998), and cytochrome C (Cyt C, AB90529) were procured from Abcam, Ltd.; and Phospho‐IRS‐1 (Tyr628) mouse/(Tyr632) (09‐433) was obtained from Sigma‐Aldrich. Protein bands were visualized using a commercially available enhanced chemiluminescence assay kit (Bio‐Rad) according to the manufacturer's instructions.

### 2.5 Annexin V/PI staining

Apoptotic cells were identified using a commercially available Annexin V‐FITC Apoptosis Detection Kit (KeyGen, Nanjing, China) according to the manufacturer's instructions. Cells were seeded into 6‐well plates at a density of 1 × 10^6^ cells per well. The cells were then washed with phosphate‐buffered saline (PBS) and harvested without EDTA. The cells in each well were resuspended in 0.5‐mL binding buffer in a 1.5‐mL tube and incubated with 5 μL of Annexin V‐FITC and 5‐μL propidium iodide for 20 minutes at room temperature (25 ± 5℃) in the dark. After staining, apoptotic cell death was assessed using fluorescence‐activated cell sorting techniques (FACSCalibur, Becton Dickinson).

### 2.6 Immunocytochemical assay

Cells were rinsed with PBS, then incubated in 4% buffered paraformaldehyde solution (pH 7.4) for 20 minutes at room temperature (25 ± 5℃). The cells were then seeded in 6‐well plates and washed with PBST at least three times. After blocking with 3% bovine serum albumin in PBST for 1 hour, the cells were treated with anti‐Bcl‐2 antibody overnitght at 4℃ and then washed with PBST. Donkey anti‐rabbit IgG H&L (Alexa Fluor® 488; Yeasen, Shanghai, China) was added to the cells and incubated for 1 hour. Mitochondria were identified by Mito red (KeyGen, Nanjing, China) according to the manufacturer's instructions. When staining for nuclei, the medium was removed and PBST was added to wash the cells gently three times before exposure to DAPI for 15 minutes. After rinsing with PBS, the cells were visualized using a laser scanning confocal microscope (FV10‐ASW, Version 2.1, MPE FV1000, Olympus Corp).

### 2.7 Scanning electron microscopy

HCT116 cells were seeded and treated with 2.5% glutaraldehyde solution at 4°C for 2 hours. The cells were then dehydrated, embedded in paraffin, and sliced to observe the ultrastructure of mitochondria. This experiment was performed at the electron microscopy laboratory of the Zhongda Hospital Southeast University (Nanjing, Jiangsu, China).

### 2.8 Statistical analysis

The results are representative of three independent experiments and expressed as mean ± SEM. Statistical analysis was performed using Prism version 6.0 (GraphPad). Comparisons were performed using the Student's *t* test (two‐tailed) and differences with *P* < .05 were considered to be statistically significant.

## 3 RESULTS

### 3.1 Insulin‐induced oxaliplatin resistance in HCT116 cells

**FIGURE 1:**
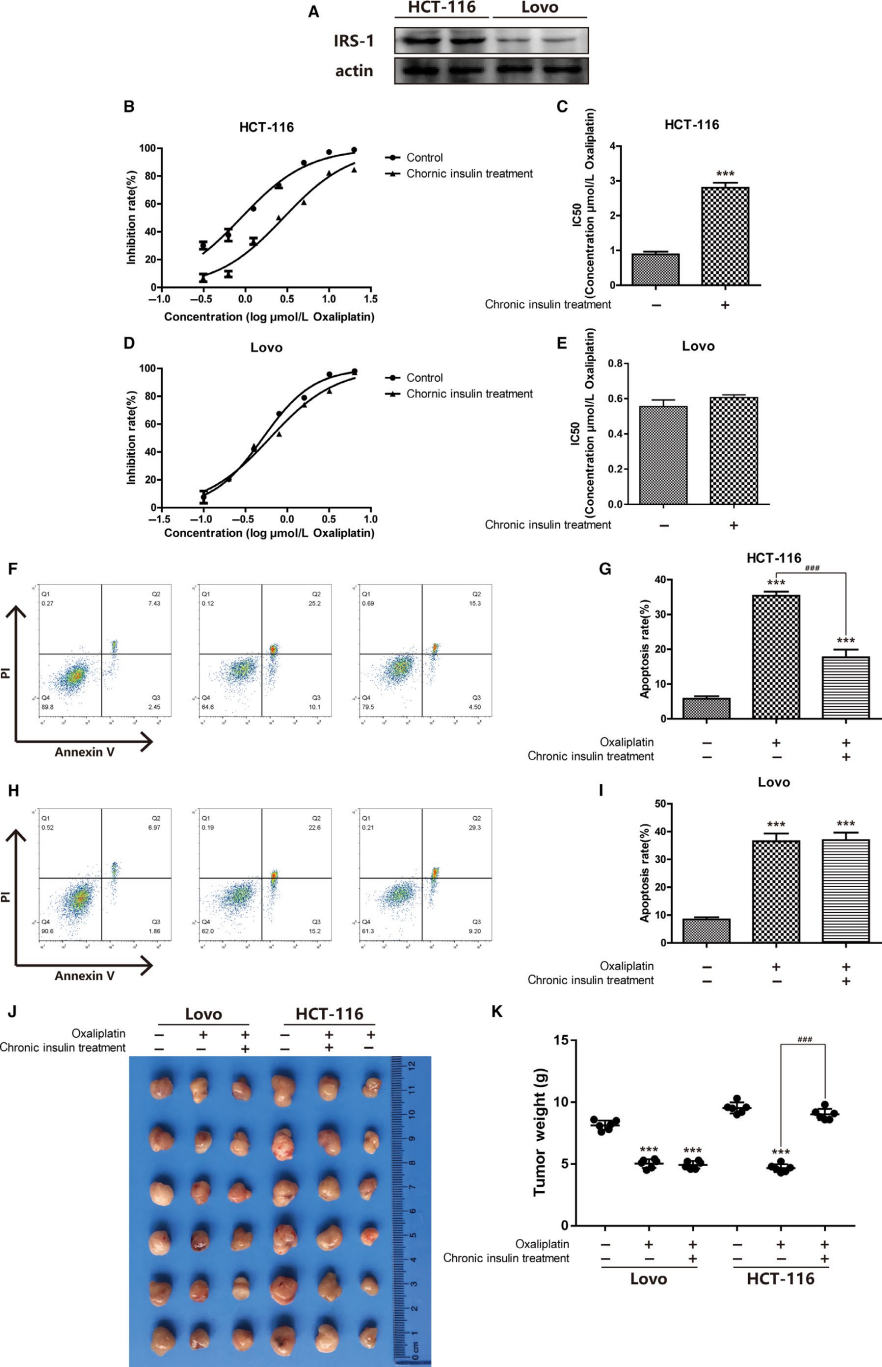
Insulin induce oxaliplatin resistance in HCT116 cells. A, HCT116 cells were incubated, then cell lysates were collected to proceed immunoblot analysis. IRS‐1 expression was detected by western blot as described. B‐C, Inhibition rate of HCT116 cells were assessed after 20 nmol/L 12 wks of insulin stimulation by CCK‐8 assay. Bars represent SEM, ^***^ *P* < .005 vs nontreated control of HCT116 cells. D‐E, Inhibition rate of LoVo cells were assessed after 20 nmol/L 12 wks of insulin stimulation by CCK‐8 assay. Bars represent SEM, ^***^ *P* < .005 vs nontreated control of LoVo cells. F‐G, HCT116 cells were stained with Annexin V and PI and apoptosis cells were quantitated by flow cytometer after chronic insulin treatment. Results from the experiments are shown as means ± SEM. The data are presented as percentage of cell apoptotic rate to unstimulated cells (0 μmol/L), ^***^ *P* < .005 vs nontreated control of HCT116 cells, ^###^ *P* < .005 vs chronic insulin treatment HCT116 cells. H‐I, LoVo were stained with Annexin V and PI and apoptosis cells were quantitated by flow cytometer after chronic insulin treatment. Results from the experiments are shown as means ± SEM. The data are presented as percentage of cell apoptotic rate to unstimulated cells (0 μmol/L), ^***^ *P* < .005 vs nontreated control of LoVo cells. J‐K, LoVo and HCT116 tumors were transplanted into BALB/C nude mice. Bars represent SEM, ^***^ *P* < .005 vs nontreated control of LoVo and HCT116 cells

Insulin resistance may contribute to chemotherapy resistance in individuals with colon cancer; as such, two colon cancer cell lines were used to determine whether the expression of IRS‐1 would affect the half maximal inhibitory concentration (IC50) of oxaliplatin after chronic insulin treatment (Figure 1A). The IC50 of oxaliplatin was tested after cells were exposed to 20 nmol/L insulin for 12 weeks. Results suggested that the tolerance of HCT116 cells to oxaliplatin was enhanced, and that IRS‐1 expression was higher than that in LoVo (Figure 1B,C). The tolerance of LoVo cells barely varied after insulin was added (Figure 1D,E). The apoptosis rates of cells treated with oxaliplatin also confirmed that long‐term exposure to insulin could alter oxaliplatin sensitivity in HCT116 cells. Such variation may be triggered by IRS‐1 phosphorylation as well as activation of downstream signaling (Figure 1F‐I). A tumor xenograft model in BALB/C nude mice was used to verify insulin‐induced oxaliplatin resistance in vivo, and the results agreed with those from the in vito experiments (Figure 1J,K).

### 3.2 Insulin‐induced oxaliplatin resistance via the mitochondrial apoptosis pathway

**FIGURE 2:**
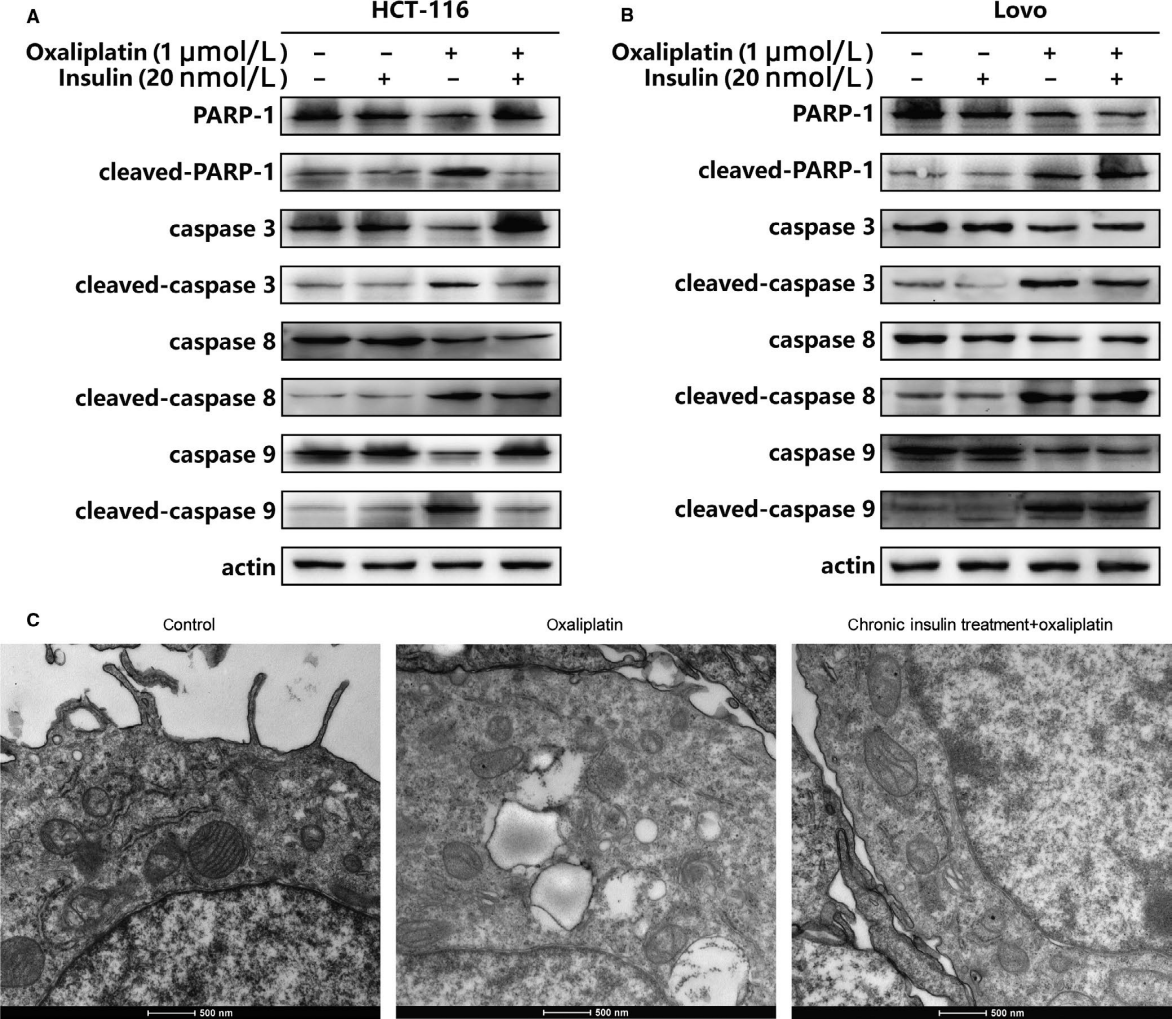
Insulin induce oxaliplatin resistance via mitochondrial apoptosis pathway. A, HCT116 cells were incubated with 20 nmol/L 12 wks of insulin stimulation, then cell lysates were collected to proceed immunoblot analysis. RARP‐11, caspase‐3, caspase‐8, caspase‐9, and their cleaved proteins’ expression were detected by western blot as described. B, LoVo cells were incubated, then cell lysates were collected to proceed immunoblot analysis. RARP‐11, caspase‐3, caspase‐8, caspase‐9, and their cleaved proteins’ expression were detected by western blot as described. C, Electronic microscope scan of HCT116 mitochondria after chronic insulin treatment

After confirming that chronic insulin treatment could alter the efficacy of chemotherapeutic drugs in HCT116 cells, key proteins in the apoptosis pathway were tested by western blot. Results suggested that cleaved caspase‐9 was downregulated noticeably in HCT116 cells (Figure 2A,B), indicating that the activity of the mitochondrial apoptotic pathway was reduced. The results of electron microscopy also indicated that the integrity of the mitochondria was retained. Although the mitochondria had become swollen, the crista remained clearly visible, suggesting that some mitochondrial protective mechanism may have been activated by long‐term (ie, chronic) insulin treatment (Figure 2C).

### 3.3 Metformin sensitized HCT116 cells via Bcl‐2 inhibition

**FIGURE 3:**
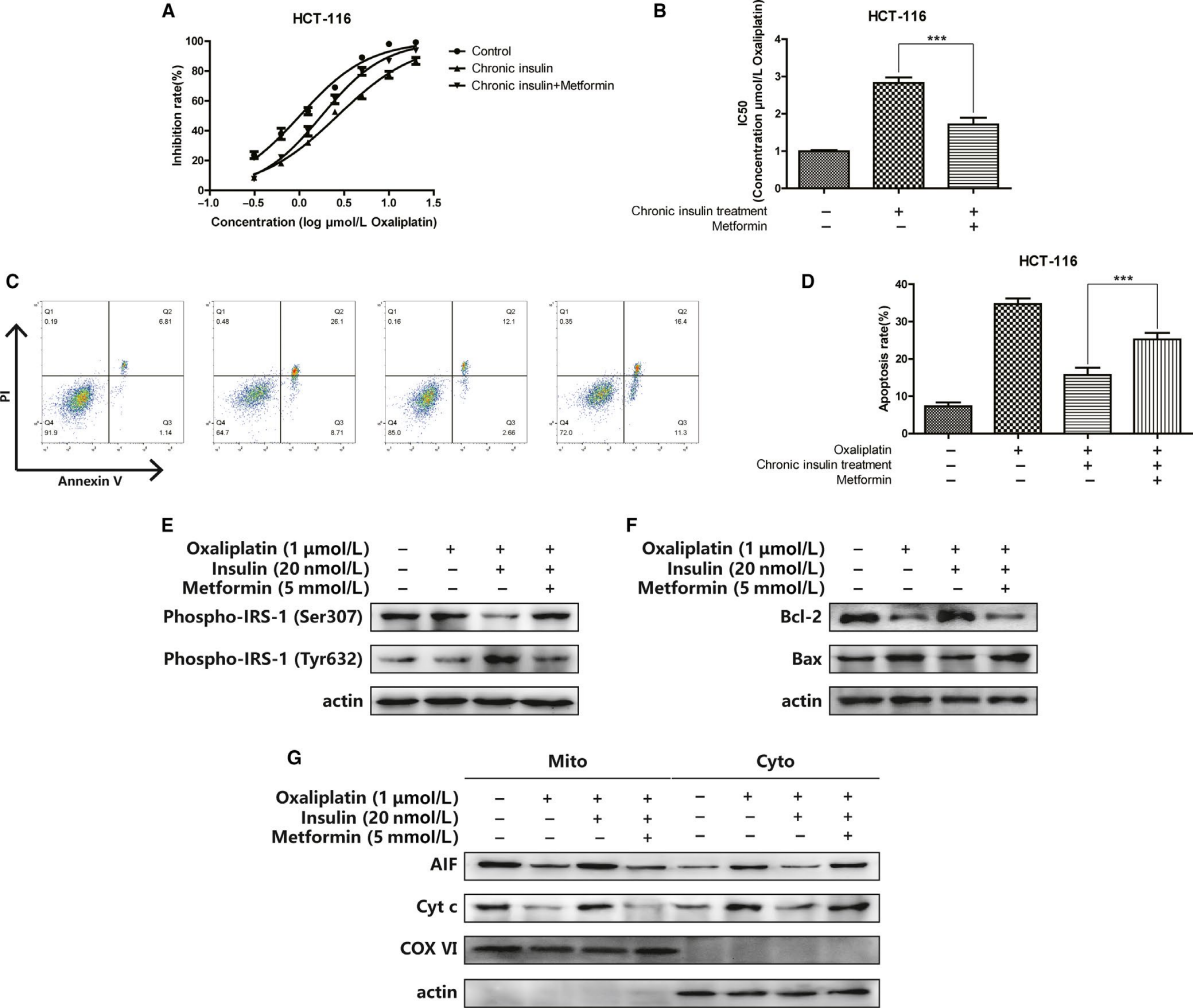
Metformin sensitize oxaliplatin in HCT116 cells via Bcl‐2 inhibition. A‐B, Inhibition rate of HCT116 cells was assessed after 20 nmol/L 12 wks of insulin stimulation combined with metformin (5 mmol/L) by CCK‐8 assay. Bars represent SEM, ^***^ *P* < .005 vs non metformin‐treated HCT116 cells C‐D, HCT116 cells were stained with Annexin V and PI and apoptosis cells were quantitated by flow cytometer after chronic insulin treatment combined with metformin (5 mmol/L). Results from the experiments are shown as means ± SEM. The data are presented as percentage of cell apoptotic rate to unstimulated cells (0 μmol/L), ^***^ *P* < .005 vs non metformin‐treated HCT116 cells. E‐G, HCT116 cells were incubated with 20 nmol/L 12 wks of insulin stimulation with metformin (5 mmol/L) combination as described, then Phospho‐IRS‐1 (Ser307), Phospho‐IRS‐1 (Tyr632), Bcl‐2, Bax expression and AIF, and Cyt C distribution in mitochondria and cytoplasm were analyzed

Metformin, an insulin sensitizer, has recently been shown to exert multiple antitumor effects. We believe that this chemotherapeutic resistance could be reversed by metformin. The IC50 of oxaliplatin was significantly reduced when metformin was added (Figure 3A,B). The apoptosis rate was also enhanced by the effect of metformin (Figure 3C,D). Results suggested that after chronic insulin treatment, the IRS‐1 phosphorylation site switched from Tyr632 to Ser307; when metformin was administered, this switch was reversed (Figure 3E). Figure 3F illustrates that the expression of Bcl‐2 was upregulated significantly after chronic insulin treatment, and this increase vanished when metformin was added. Afterward, the mitochondria of HCT116 cells were harvested, and critical proteins were tested to assess mitochondrial integrity. The results demonstrated that integrity was damaged and AIF and Cyt c leaked from the mitochondria (Figure 3G). These results revealed that the mechanism of oxaliplatin sensitization through metformin may follow Bcl‐2 inhibition.

### 3.4 Metformin inhibited Bcl‐2 expression via AMPK activation

**FIGURE 4:**
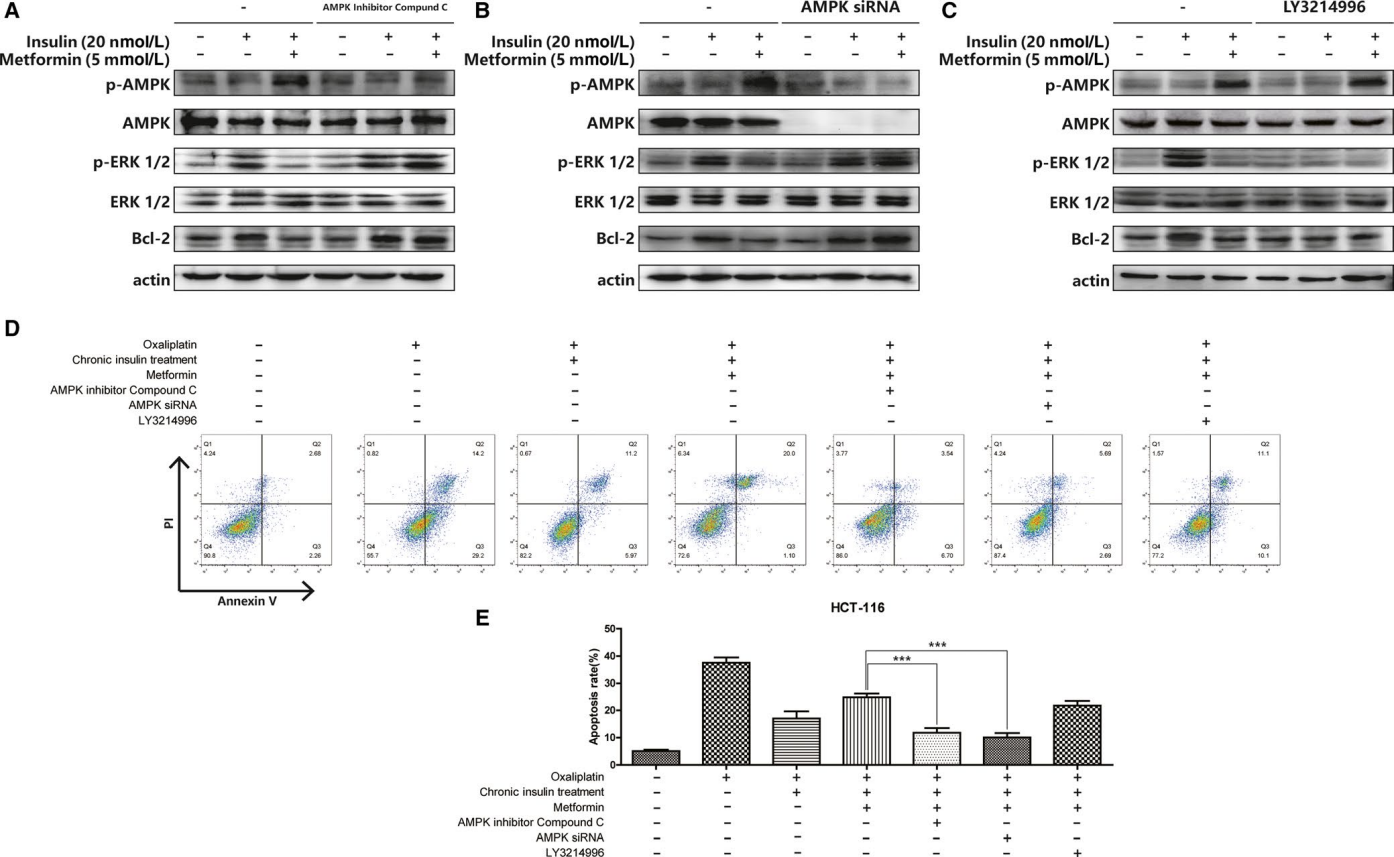
Metformin inhibit Bcl‐2 expression via AMPK activation. A‐C, HCT116 cells were incubated with 20 nmol/L 12 wks of insulin stimulation with metformin (5 mmol/L) combination as described, then AMPK inhibitor compound C, AMPK siRNA, and LY3214996 were applied as described. Then AMPK, ERK, and Bcl‐2 expression were analyzed by immunoblot. D‐E, HCT116 cells were stained with Annexin V and PI and apoptosis cells were quantitated by flow cytometer after chronic insulin treatment combined with metformin (5 mmol/L), then AMPK inhibitor compound C, AMPK siRNA, and LY3214996 were applied as described. Bars represent SEM, ^***^ *P* < .005 vs chronic insulin + metformin‐treated HCT116 cells

To examine how metformin affects Bcl‐2 expression, key proteins in the AMPK signaling axis were tested. As shown in Figure 4A, metformin activated AMPK, as well as downregulated Erk phosphorylation and Bcl‐2 expression. When the AMPK inhibitor compound C was used, AMPK phosphorylation was reduced, and inhibition of Erk and Bcl‐2 was mitigated. The same tendency in variation was observed in the expression of Erk and Bcl‐2 when AMPK activation was blocked using AMPK small interfering RNA (siRNA), and inhibition was also eliminated (Figure 4B). Next, another parallel experiment was performed using the Erk inhibitor LY3214996. The results showed that even without AMPK activation, Bcl‐2 expression was reduced when Erk phosphorylation was inhibited (Figure 4C). According to these results, metformin‐mediated Bcl‐2 inhibition followed the AMPK/Erk signaling pathway. Subsequently, the apoptosis rate was ascertained to assess the sensitization of metformin in combination with oxaliplatin. The results indicated that the increase in metformin‐induced apoptosis rate was mitigated by the AMPK inhibitor and AMPK siRNA. The apoptosis rate increased when oxaliplatin was combined with LY3214996 (Figure 4D,E). According to these results, the mechanism of metformin‐induced sensitization in oxaliplatin followed Bcl‐2 inhibition via the AMPK/Erk signaling pathway.

### 3.5 Metformin‐induced Bcl‐2 inhibition promoted apoptosis through damage to mitochondrial integrity

**FIGURE 5:**
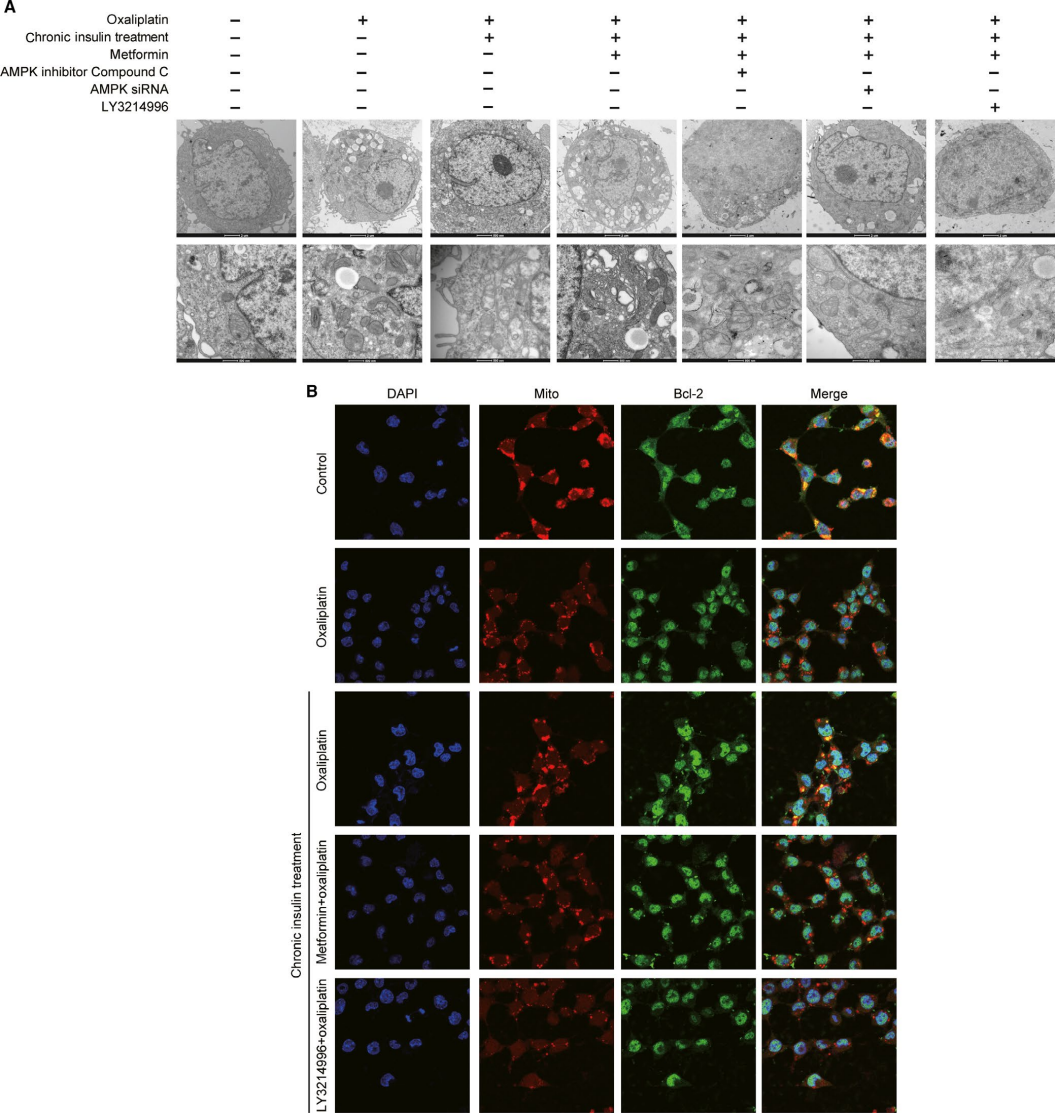
Metformin‐induced Bcl‐2 inhibition promote apoptosis though mitochondria integrity damage. A, Electronic microscope scan of HCT116 mitochondria after chronic insulin treatment with metformin combination, then AMPK inhibitor compound C, AMPK siRNA, and LY3214996 were applied as described. B, HCT116 cells were incubated with 20 nmol/L 12 wks of insulin stimulation with metformin (5 mmol/L) combination as described, then LY3214996 was applied as described. Confocal images of the cells show the fluorescence of nucleus in blue, mitochondria in red, Bcl‐2 in green, and the merged images in Column 4

Because Bcl‐2 is a critical antiapoptotic protein in the mitochondrial apoptosis pathway, it can maintain mitochondrial integrity by self‐translocation. Thus, immunofluorescence and scanning electron microscopy were performed to explore how metformin affects the mitochondria (Figure 5A,B). The results indicated that Bcl‐2 translocation was inhibited and that this inhibition process could be eliminated by AMPK deactivation. Electron microscopy also suggested that mitochondrial integrity was damaged when AMPK was activated, and such an injury effect could be reversed by Erk inhibition. These results further revealed that metformin facilitated mitochondrial apoptosis and enhanced oxaliplatin sensitivity in HCT116 cells.

## 4 DISCUSSION

Oxaliplatin is a third‐generation platinum compound that has been widely used to treat many types of cancers, including colon cancer.^10^, ^11^, ^12^ However, drug resistance often occurs throughout long‐term chemotherapy. Chemotherapy resistance has been a consistent and inevitable problem in cancer therapy since drugs were developed for clinical applications. Accordingly, numerous researchers have committed to discovering an agent or method to reverse this process.^13^, ^14^ Metformin, an oral biguanide hypoglycemia drug, has been found to exert potential anticancer effects. Metformin has also been shown to act as a chemotherapeutic sensitizer.^15^ Our research aimed to identify the underlying mechanism of how metformin induces sensitization in combined treatment.

To ascertain the chemotherapeutic sensitization effect, an oxaliplatin‐resistant cell model was built using long‐term, low‐dose, insulin treatment (20 nmol/L, 12 weeks). In this model, tolerance to oxaliplatin was enhanced noticeably, suggesting that long‐term exposure to high levels of insulin, in fact, worsens patient prognosis. It has been established that tolerance to oxaliplatin increases after insulin treatment; more specifically, the apoptosis signaling pathway may be hindered in this process. Thus, several key proteins involved in the classic apoptosis signaling pathway were tested by western blotting, and it was found that Bcl‐2, which is involved in the mitochondrial protective mechanism, was activated by insulin.

The Bcl‐2 protein family has been well demonstrated to be associated with mitochondrial function, especially in maintaining mitochondrial permeability by mitochondrial translocation. Metformin is capable of inhibiting cancer cell proliferation by Bcl‐2 inhibition^16^; as such, metformin may reverse insulin‐induced oxaliplatin resistance in HCT116 cells. Given that hyperinsulinemia is likely to cause drug resistance, metformin can lead to variations in the phosphorylation position of IRS‐1 to evoke insulin sensitivity; thus, this shift may be critical to metformin‐mediated sensitization of oxaliplatin. Metformin induces its pharmacodynamic action primarily by activating AMPK, and two types of phosphorylation of IRS participate in AMPK activation and deactivation, namely Tyr632 and Ser307.^17^ Accordingly, the shift in the phosphorylation position of IRS‐1 may be critical to the variation in oxaliplatin sensitivity.

There are numerous downstream signaling pathways of AMPK, among which Erk inhibition may be a cause of Bcl‐2 inhibition. It has been reported that human melanoma cell apoptosis could be induced by negatively regulating the Erk/PKM2/Bcl‐2 axis.^18^ Metformin could enhance the inhibition of Erk through AMPK activation in combination with gefitinib^19^; therefore, metformin‐regulated Bcl‐2 inhibition may occur via the AMPK/Erk signaling pathway. To test our hypothesis, AMPK phosphorylation and Erk phosphorylation were blocked separately, and then changes in the pattern of Bcl‐2 expression were analyzed. These results confirmed our hypothesis, suggesting that metformin can inhibit Bcl‐2 expression by AMPK activation via Erk dephosphorylation. Bcl‐2 is a critical antiapoptotic protein in the mitochondrial apoptosis pathway and can maintain mitochondrial integrity by self‐translocation in the mitochondria. According to the results of immunofluorescence and electron microscopy, metformin destroyed the structure of mitochondria by inhibiting Bcl‐2 mitochondrial translocation.

## 5 CONCLUSION

Our studies involving chronically insulin‐treated HCT116 cells suggested that metformin could inhibit Bcl‐2 expression and its mitochondrial translocation, thereby facilitating mitochondrial apoptosis via the AMPK/Erk signaling pathway. Metformin is likely to mitigate oxaliplatin‐mediated apoptosis by altering the IRS‐1 phosphorylation site from Ser307 to Tyr632 and amplifying Erk dephosphorylation via AMPK activation. This further demonstrated the sensitization of metformin combined with oxaliplatin. Results of this study should prompt clinicians and researchers to pursue the promising potential of metformin as a novel agent of clinical interest in the therapy of colon cancer patients who also have type II diabetes.

## CONFLICT OF INTEREST

The authors declare that they have no competing interests.

## AUTHOR CONTRIBUTIONS

Chen Qiao and Qianming Du conceived and designed the study. Qianqian Liu, Aiwen Yan, Yuyin Ding, and Junye Tao performed the experiments. Chen Qiao wrote, reviewed, and edited the manuscript. All authors read and approved the final manuscript.

## Data Availability

The data used to support the findings of this study are available from the corresponding author upon request.

## References

[1] Siegel RL , Miller KD , Jemal A . Cancer statistics, 2017. CA Cancer J Clin. 2017;67:7‐30. 28055103 10.3322/caac.21387

[2] Huang Y‐C , Lin J‐K , Chen W‐S , et al. Diabetes mellitus negatively impacts survival of patients with colon cancer, particularly in stage II disease. J Cancer Res Clin Oncol. 2011;137:211‐220. 20387072 10.1007/s00432-010-0879-7 PMC11828289

[3] Baricevic I , Roberts DL , Renehan AG . Chronic insulin exposure does not cause insulin resistance but is associated with chemo‐resistance in colon cancer cells. Horm Metab Res. 2014;46:85‐93. 24068609 10.1055/s-0033-1354414

[4] Wolpin BM , Meyerhardt JA , Chan AT , et al. Insulin, the insulin‐like growth factor axis, and mortality in patients with nonmetastatic colorectal cancer. J Clin Oncol. 2009;27:176‐185. 19064975 10.1200/JCO.2008.17.9945 PMC2645084

[5] Dowling RJ , Zakikhani M , Fantus IG , Pollak M , Sonenberg N . Metformin inhibits mammalian target of rapamycin‐dependent translation initiation in breast cancer cells. Cancer Res. 2007;67:10804‐10812. 18006825 10.1158/0008-5472.CAN-07-2310

[6] Mallik R , Chowdhury TA . Metformin in cancer. Diabetes Res Clin Pract. 2018;143:409‐419. 29807101 10.1016/j.diabres.2018.05.023

[7] Cheng Y , Chen Y , Zhou C , et al. For colorectal cancer patients with type II diabetes, could metformin improve the survival rate? A meta‐analysis. Clin Res Hepatol Gastroenterol. 2020;44(1):73‐81. 31300371 10.1016/j.clinre.2019.06.009

[8] Hemkens LG , Grouven U , Bender R , et al. Risk of malignancies in patients with diabetes treated with human insulin or insulin analogues: a cohort study. Diabetologia. 2009;52:1732‐1744. 19565214 10.1007/s00125-009-1418-4 PMC2723679

[9] Huang XU , Wullschleger S , Shpiro N , et al. Important role of the LKB1‐AMPK pathway in suppressing tumorigenesis in PTEN‐deficient mice. Biochem J. 2008;412:211‐221. 18387000 10.1042/BJ20080557

[10] Fountzilas E , Krishnan E , Janku F , et al. A phase I clinical trial of hepatic arterial infusion of oxaliplatin and oral capecitabine, with or without intravenous bevacizumab, in patients with advanced cancer and predominant liver involvement. Cancer Chemother Pharmacol. 2018;82:877‐885. 30182147 10.1007/s00280-018-3680-y

[11] Papaxoinis G , Kotoula V , Giannoulatou E , et al. Phase II study of panitumumab combined with capecitabine and oxaliplatin as first‐line treatment in metastatic colorectal cancer patients: clinical results including extended tumor genotyping. Med Oncol. 2018;35:101. 29855806 10.1007/s12032-018-1160-1

[12] Shen Y , Li C , Liu W , et al. Clinical analysis of hypersensitivity reactions to oxaliplatin among colorectal cancer patients. Oncol Res. 2018;26:801‐807. 29295722 10.3727/096504017X15139039328978 PMC7844702

[13] Shen L , et al. beta3GnT8 regulates oxaliplatin resistance by altering integrin beta1 glycosylation in colon cancer cells. Oncol Rep. 2018;39:2006‐2014. 29393491 10.3892/or.2018.6243

[14] Lai HH , Lin LJ , Hung LY , Chen PS . Role of dicer in regulating oxaliplatin resistance of colon cancer cells. Biochem Biophys Res Commun. 2018;506:87‐93. 30336979 10.1016/j.bbrc.2018.10.071

[15] Banerjee A , Birts CN , Darley M , et al. Stem cell‐like breast cancer cells with acquired resistance to metformin are sensitive to inhibitors of NADH‐dependent CtBP dimerization. Carcinogenesis. 2019;40(7):871‐882. 30668646 10.1093/carcin/bgy174

[16] Sharma P , Kumar S . Metformin inhibits human breast cancer cell growth by promoting apoptosis via a ROS‐independent pathway involving mitochondrial dysfunction: pivotal role of superoxide dismutase (SOD). Cell Oncol (Dordr). 2018;41:637‐650. 30088260 10.1007/s13402-018-0398-0 PMC12995255

[17] Liu K‐L , Kuo W‐C , Lin C‐Y , et al. Prevention of 4‐hydroxynonenal‐induced lipolytic activation by carnosic acid is related to the induction of glutathione S‐transferase in 3T3‐L1 adipocytes. Free Radic Biol Med. 2018;121:1‐8. 29698741 10.1016/j.freeradbiomed.2018.04.567

[18] Zhao H , et al. Resveratrol induces apoptosis in human melanoma cell through negatively regulating Erk/PKM2/Bcl‐2 axis. Onco Targets Ther. 2018;11:8995‐9006. 30588012 10.2147/OTT.S186247 PMC6294058

[19] Peng M , Huang Y , Tao T , et al. Metformin and gefitinib cooperate to inhibit bladder cancer growth via both AMPK and EGFR pathways joining at Akt and Erk. Sci Rep. 2016;6:28611. 27334428 10.1038/srep28611 PMC4917871

