# Corrigendum

**DOI:** 10.1002/cam4.3702

**Published:** 2021-03-02

In the article by Chao Liu et al., the corresponding author would like to include the affiliation for Qianqian Liu in author byline and also add the authors contribution line.

The included affiliation and authors contribution line should read as follows:

Metformin Revert Insulin‐induced Oxaliplatin Resistance by Activating Mitochondrial Apoptosis Pathway in Human Colon cancer HCT116 Cells

Chao Liu^1,#^, Qianqian Liu^2,#^, Aiwen Yan^1^, Hui Chang^1^, Yuyin Ding^1^, Junye Tao^1^, Chen Qiao^1,*^

^1^Department of Pharmacy, Nanjing First Hospital, China Pharmaceutical University, Nanjing, Jiangsu, China

^2^China Pharmaceutical University, School of Basic Medicine and Clinical Pharmacy, Nanjing, 211198, China

^#^Chao Liu and Qianqian Liu are contributed equally to this work.

The authors have also noticed an error that the western blot band was misplaced in Figure 4. The correct version of the Figure 4 is displayed below.

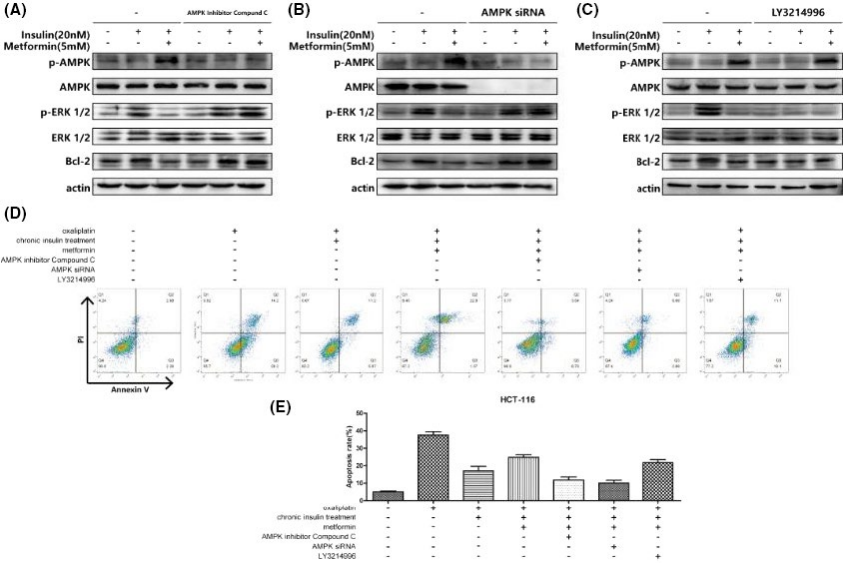

**FIGURE 4** Metformin inhibit Bcl‐2 expression via AMPK activation. A‐C, HCT116 cells were incubated with 20 nmol/L 12 wks of insulin stimulation with metformin (5 mmol/L) combination as described, then AMPK inhibitor compound C, AMPK siRNA, and LY3214996 were applied as described. Then AMPK, ERK, and Bcl‐2 expression were analyzed by immunoblot. D‐E, HCT116 cells were stained with Annexin V and PI and apoptosis cells were quantitated by flow cytometer after chronic insulin treatment combined with metformin (5 mmol/L), then AMPK inhibitor compound C, AMPK siRNA, and LY3214996 were applied as described. Bars represent SEM, ***P < .005 vs chronic insulin + metformin‐treated HCT116 cells

The authors apologize for this error.
